# Experiences of Nursing Students Regarding Challenges and Support for Resilience during Clinical Education: A Qualitative Study

**DOI:** 10.3390/nursrep14030120

**Published:** 2024-06-28

**Authors:** Pimwalunn Aryuwat, Jessica Holmgren, Margareta Asp, Matanee Radabutr, Annica Lövenmark

**Affiliations:** 1 School of Health, Care and Social Welfare, Mälardalen University, 721 23 Vasteras, Sweden; jessica.holmgren@mdu.se (J.H.); margareta.asp@mdu.se (M.A.); annica.lovenmark@mdu.se (A.L.); 2 Boromarajonani College of Nursing, Changwat Nonthaburi, Faculty of Nursing, Praboromarajchanok Institute, Nonthaburi 11000, Thailand; matanee@bcnnon.ac.th

**Keywords:** challenges, clinical education, nursing education, nursing students, resilience, support, thematic analysis

## Abstract

Nursing students require resilience to navigate the complexities of clinical practice. This characteristic is essential for managing demanding workloads, unpredictable patient situations, and emotional stressors while maintaining performance and well-being. Fostering resilience helps students develop the capacity to adapt to adversity, overcome setbacks, and remain committed to providing high-quality patient care. This qualitative study explores the challenges and supports influencing nursing students’ resilience during clinical education. Interviews with 28 Thai nursing students revealed two key themes: the “experience of vulnerability” and the “experience of meaningfulness”. The sub-themes of vulnerability included “navigating uncertainty”, “transcending professional struggles”, and “being exposed to diverse encounters”. The sub-themes of meaningfulness focused on restoring strength through social interactions and engaging in positive transformation. This study highlights the need for comprehensive support systems that address personal and professional vulnerabilities. Integrating caring theory principles could further enhance resilience by emphasizing compassionate care and fostering student empathy. This suggests that instructors and stakeholders can significantly impact student well-being by creating supportive environments built on collaboration, empathy, and mentorship, all of which are aligned with caring theory.

## 1. Introduction

Engaging in the field of nursing involves confronting formidable challenges [1]. The educational journey in nursing is characterized by its demanding nature, involving intricate coursework, clinical training, and rigorous examinations [2]. Resilience is a crucial attribute for nursing students to successfully navigate these challenges, fostering both personal and professional development [3]. The adept handling of challenges is highly esteemed in nursing education, where critical thinking skills are imperative for prompt and effective decision-making [4,5]. The intricate relationship between resilience and self-care practices is a significant aspect of nursing education. Fostering the connection between resilience and self-care practices is essential for preparing nursing students to manage the demands of the nursing profession effectively [6]. Resilient nursing students exhibit a propensity to prioritize their well-being, a practice that holds potential advantages in maintaining an optimal work-life balance, averting exhaustion, and nurturing satisfaction within the nursing profession [7].

The demanding nature of nursing education, coupled with the emotional complexities of caring for patients, necessitates the development of resilience in nursing students. The positive influence of resilience on mitigating academic burnout and psychological distress among nursing students during challenging circumstances has been highlighted [8,9]. This aligns with the core tenets of caring science, which emphasize fostering compassion and well-being in patients and caregivers. Integrating resilience-building strategies within nursing education enables nursing instructors to cultivate the emotional strength and adaptability required for nursing students to navigate the demanding realities of the profession while upholding the core values of caring and empathy [10]. There is a critical need to explore the potential benefits of integrating human caring theory with evidence-based principles to foster resilience in nursing students [11]. Unitary caring science, the foundation of Jean Watson’s theory of human caring, offers a valuable framework for nursing practice [12]. Jean Watson’s theory of human caring, particularly the unitary caring science perspective, provides a valuable framework for understanding and fostering resilience in nursing students [10,12]. The core principles of this theory, such as the cultivation of caring relationships, the promotion of holistic well-being, and the emphasis on self-care practices, align closely with the concept of resilience [10]. Exploring the resilience of nursing students through the lens of Jean Watson’s theory of human caring allows for a more comprehensive understanding of the factors that contribute to resilience development. Considering the role of caring practices, supportive relationships, and holistic well-being in the context of clinical education allows for the identification of challenges and supportive strategies that can serve as baseline information for enhancing resilience and better preparing nursing students for the demands of the profession.

Clinical practice constitutes an indispensable component of nursing education, serving as a crucible where students encounter the application of theoretical knowledge and challenging encounters that test their courage in real-life situations [13]. This juncture emerges as a critical period for nursing students, in which they grapple with significant adversities, rendering resilience an essential facet contributing to their efficient transition [14]. The challenges encountered during clinical practice serve as catalysts for honing critical thinking skills, decision-making abilities, and emotional resilience, cultivating adaptability, and fostering a sense of professional competence [15]. However, adequate support is equally indispensable for developing resilience [16]. A supportive learning environment characterized by effective supervision, constructive feedback, and a culture of collaboration strengthens coping mechanisms, enhances learning experiences, and contributes to the development of resilient and proficient practitioners [17,18,19]. The initial critical stage in nursing students’ transition into nursing has garnered extensive attention, particularly in clinical education [20,21,22,23]. Discovering the complex tapestry of nursing students’ encounters and their resilience during clinical practice becomes a scholarly quest and a pragmatic necessity. This comprehension is vital for optimizing educational strategies, mentorship programs, and support mechanisms to enhance nursing students’ resilience during clinical practice [8,16]. Exploring resilience in this context enables the cultivation of an educational environment that not only equips nursing students with the requisite clinical skills but also supports their capacity to navigate the complexity of challenges characterizing their initial entry into the nursing profession.

Resilience is universally acknowledged as an indispensable facet of positive health [8,24,25]. In nursing education, resilience is explicitly characterized as the capacity to manage stressors effectively [2,26]. Recent conceptualizations of resilience within the nursing student population elucidate it as a dynamic process integral to personal development [8]. This process is closely linked to protective factors that facilitate the adept handling of stressors and adversities [27,28]. Transition phases, such as the progression from adolescence to adulthood and the shift from theoretical learning to clinical training, introduce numerous challenges for nursing students. The imperative to adapt to changes inherent in these transitional periods may subject nursing students to extensive pressure, thus posing potential threats to their overall health, particularly their mental well-being [24,25]. A heightened resilience emerges as a distinct advantage for adolescents navigating the challenges and responsibilities accompanying the transition to adulthood, especially when confronted with adverse circumstances [24]. Moreover, the significance of resilience extends to undergraduate and graduate nursing students, highlighting its vital role in their educational journeys [29].

Predominantly, global research into the resilience of nursing students has been centered on exploring the connection between resilience and mental health, as well as overall well-being [30,31]. The trial of clinical practice exposes nursing students to a spectrum of new challenges, including assimilating into the distinctive academic culture of higher education, assuming responsibility for patient care, navigating the weighty expectations associated with academic excellence, and proficiently managing assignments within stipulated deadlines [31,32]. Consequently, there is a discernible need to comprehend the conceptualization of resilience among nursing students in the specific milieu of academic learning, with a pronounced emphasis on its manifestation during clinical practice. Such insights hold practical value for the advancement of nursing education [8,33].

While the importance of resilience in nursing education is well-established, there is a paucity of comprehensive studies specifically addressing the challenges and support for resilience experienced by nursing students in clinical settings. This knowledge gap impedes the development of effective evidence-based interventions tailored to the unique demands of clinical practice among nursing students [8,33]. The current study focuses on clinical education, as it adds a layer of complexity to the already demanding nature of nursing education. The stressors and challenges encountered in the clinical setting differ significantly from those in the classroom, and they can have a significant impact on students’ learning and professional development [34]. This research specifically focuses on the clinical aspect of nursing education. This choice was made to address the unique challenges faced by nursing students in clinical settings, which differ significantly from those encountered in didactic learning environments. While didactic education primarily deals with theoretical knowledge and classroom learning, clinical education involves real-world patient care, requiring students to apply theoretical knowledge in practical, often high-pressure, situations [35]. This distinction is crucial because the stressors associated with clinical education, such as direct patient interaction, handling medical emergencies, and managing clinical workload, necessitate different coping strategies and support mechanisms. Exploring the resilience of nursing students in the context of clinical practice provides valuable insights for those seeking to develop strategies to support and enhance resilience in this specific aspect of nursing education.

## 2. Materials and Methods

### 2.1. Aim

The aim of this study was to describe nursing students’ experiences regarding the challenges and support for resilience within the context of clinical education.

### 2.2. Study Design

In this study, we utilized a qualitative approach to obtain comprehensive insights. Semi-structured individual interviews were conducted meticulously to acquire in-depth data. Subsequently, thematic analysis was employed to analyze the gathered information.

### 2.3. Procedure and Participants

The participants in this study included 28 nursing students from three nursing colleges under the management of the Faculty of Nursing at the Praboromarajchanok Institute (PBRI) in Thailand. All nursing colleges within the jurisdiction of the PBRI adhere to the same curriculum and operate within the same standardized academic system. The recruitment process involved the solicitation of volunteers. Eligible participants were defined as those currently engaged as full-time students pursuing a Bachelor of Nursing degree with recent completion of their clinical practice experiences. Data collection commenced after the attainment of all requisite ethical clearances. Additionally, the participants were given unequivocal assurances regarding the confidentiality of their involvement and the stringent preservation of data confidentiality throughout the investigation.

### 2.4. Data Collection and Analysis

Due to COVID-19 safety protocols, the initially planned face-to-face interviews underwent a strategic transformation, transitioning to digital platforms, specifically Zoom and Google Meet, according to the participants’ preferences. The procedural sequence unfolded as follows: (1) An official communication, in the form of a research permission request, was dispatched to the directors of the three nursing colleges constituting the research settings. (2) Following this, official approval for utilizing digital interview methods was secured from all three research settings. (3) A digital meeting with gatekeepers, specifically nursing instructors from each research setting, was organized to clarify the recruitment and data collection processes. (4) Upon obtaining endorsement from the gatekeepers, the research project received permission, and promotional materials, informational sheets, and consent forms were disseminated to eligible participants within their respective research settings. (5) Nursing students’ expressed interest in participating was communicated to the gatekeepers at their nursing colleges. (6) Volunteers for the study, having provided informed consent, were identified, and interview schedules were coordinated and communicated to the participants. (7) The link to the selected digital platform was circulated to all gatekeepers, who in turn disseminated it to the participants through diverse channels, such as email and various social media platforms (e.g., LINE, Messenger, and WhatsApp). (8) Individual in-depth interviews were conducted digitally using semi-structured interview guide to ensure consistency. (9) Subsequently, all interviews were meticulously recorded and transcribed verbatim by the researcher.

The interview guide (Table 1) were formulated with primary inquiries and exploratory questions designed to assist the researcher in maintaining focus on the study’s objectives. The interview questions encompassed a comprehensive spectrum of nursing students’ encounters with challenges and support that influenced their resilience during clinical practice. The credibility and trustworthiness of the process were established during three pilot interviews. The participants in the pilot interview were excluded from the study’s final sample.

Data analysis was performed using Braun and Clarke’s inductive thematic analysis approach (2006, 2022) [36,37]. This method was selected to facilitate a rigorous and systematic data analysis, ensuring data saturation and the meaningful categorization of themes related to challenges and support for resilience among nursing students in clinical education. The inductive approach allowed the researchers to explore the data without imposing preconceived ideas, promoting reflexivity and the awareness of inherent biases. The analysis adhered to Braun and Clarke’s six-phase process: data familiarization, initial code generation, theme search, theme review, theme definition and naming, and report production. The primary researcher investigated the data by repeatedly reading the transcripts and noting initial observations. Codes were then generated to identify the relevant features of the data. These codes were organized into potential themes, which were subsequently reviewed and refined to ensure an accurate representation of the data. The themes were then clearly defined and named to capture the essence of each theme. Finally, a comprehensive report was produced, supported by relevant data extracts. Throughout the analysis, the primary researcher engaged in regular discussions with all authors to ensure the credibility and trustworthiness of the findings. The experiences of nursing students regarding challenges and support for resilience during clinical practice remained the central focus of the analysis, ensuring that the themes were firmly grounded in the data.

Data saturation was achieved within 28 interviews, with sessions lasting between 45 and 90 min. The participant group consisted of 24 females and 4 males, aged 20 to 29 years, all in their third year of nursing studies and had completed their clinical practicum. Each participant was assigned a unique identifier using a number coding system to maintain participant anonymity (e.g., P1 for the first participant, P2 for the second participant, etc.). The data underwent a systematic coding and categorization process. Initial decontextualization isolated the narrative text from its context, allowing for independent coding to generate categories and themes. The texts were then re-evaluated based on the identified code units. Selective text highlighting facilitated theme comparison and the presentation of preliminary findings. Each theme was explored, with specific quotes emphasizing significant aspects. Through an inductive coding approach, themes were derived, and codes were clustered into significant categories for each participant’s narrative. Subsequent data reanalysis prompted a revision of the themes. The revised findings were discussed, and approval was obtained from all authors for the final analytical version. The trustworthiness and confirmability of the analysis were addressed, and participants’ quotations were presented under their respective thematic headings.

### 2.5. Ethical Considerations

Adherence to ethical standards was paramount throughout the study, aligning with the principles outlined in the Declaration of Helsinki (2013) [38]. Ethical approval for this study was obtained from the Ethics Review Authority in Sweden, under approval number 2021-03754, and from the Srimasarakham Nursing College, serving as a proxy for the Faculty of Nursing at the Praboromarajchanok Institute, Thailand, under approval number 021-2021.

## 3. Results

Two themes and five sub-themes were identified, as presented in Table 2. The experience of vulnerability encapsulated three distinct sub-themes: “navigating uncertainty” highlighted the students’ grappling with knowledge limitations and uncertainties amid the complexities of clinical settings; “transcending professional struggles” captured their feelings of inadequacy and hesitation when confronted with challenging situations; and “being exposed to diverse encounters” reflected their vulnerability to emotional and psychological stress due to the demanding nature of clinical practice. In contrast, the experience of meaningfulness presented two uplifting sub-themes: “restoring strength through social interactions” emphasized the students’ diverse sources of guidance, encouragement, and emotional support, and “engaging in positive transformation” highlighted their sense of accomplishment and personal growth through overcoming challenges and witnessing their clinical skills and confidence improve.

### 3.1. Experiences of Vulnerability

During clinical practice, nursing students encounter numerous challenges that evoke a sense of vulnerability. They are thrust into close encounters with human suffering within the clinical environment, confronting the realities of illness, injury, and mortality. They may grapple with their own limitations and insecurities as they navigate these complex situations. This clinical experience elicits a range of emotions, from apprehension and self-doubt to empathy and resilience. Nursing students may confront uncertainties, struggle with professional responsibility, and face several challenges through these encounters.

#### 3.1.1. Navigating Uncertainty

Entering clinical practice for the first time can be daunting for nursing students, particularly when confronted with uncertainties. A lack of knowledge can manifest in various ways, depending on the context and situation. Students may lack answers to nursing instructors’ questions, be unsure of their skills, or feel utterly unfamiliar with the complexities of clinical practice. Facing these uncertainties can lead to a range of negative emotions, as reported by the nursing students themselves.

One participant reflected on a pre-conference experience marked by her inability to address a nursing instructor’s inquiry concerning patient care. This lapse in knowledge induced an emotional response characterized by an acute sense of vulnerability and self-doubt regarding her competencies, contrasting starkly with her initial perception of being thoroughly prepared:

*That day during the pre-conference, when I failed to answer the nursing instructor’s question about caring for patients under my care, I was feeling like I would collapse in front of my nursing instructor and my friends. I had a feeling of self-doubting about my abilities. I really thought that I was quite well-prepared, but obviously, I was not prepared enough. I must admit that it was an embarrassing experience, and it made me feel ashamed.*

(P13)

A lack of awareness regarding their own skills poses a challenge to nursing students’ resilience. One participant reflected on her initial clinical practice, describing the experience as a “roller-coaster”, where moments of confidence swiftly transitioned into phases of pronounced uncertainty about her knowledge:

*If I have to compare the first clinical practice experience with something, I will say…(thinking)…riding on a roller-coaster, perhaps. One moment, I was so confident with my nursing skills, and the next moment, I was unsure about what I was doing. Am I doing it properly? Did I understand patient’s condition correctly? I think it is one of factors that can shake my resilience.*

(P11)

Additional instances involved another participant confronting challenges to her resilience in situations of unfamiliarity, particularly in not possessing sufficient knowledge about clinical practice. This lack of knowledge extended to a limited understanding of potential scenarios to be encountered, whether independently or in collaboration with various stakeholders, including patients, their relatives, instructors, or staff members:

*As a nursing student…and having the first clinical practice, I felt like floating in the big ocean that I may come across a ton of uncertain situations. …For example, about the patients, each patient has her own uniqueness, so ten patients would come with 10 different challenges, I felt like that.*

(P8)

In the quote above, the participant characterized her initial clinical practice as navigating “a big ocean”, where she felt adrift. This metaphor underscores the pervasive uncertainty and challenges within the clinical setting. Confronted with numerous patients, each with distinct circumstances, hopes, and needs, she found it difficult to anticipate or comprehend the array of situations she encountered. For individuals with limited or no prior clinical experience, the absence of foresight regarding expectations and potential encounters becomes a formidable challenge in maintaining or developing resilience.

#### 3.1.2. Transcending Professional Struggles

In the context of clinical practice, nursing students are expected to perform specific nursing procedures mandated for their clinical evaluation. This challenge extends beyond executing specific nursing procedures, such as blood extraction and nasogastric tube insertion, to include managing essential administrative tasks, such as the timely submission of nursing care plans. Furthermore, the reluctance to act—often attributed to fatigue—further compounds the challenges faced by nursing students. The emotional consequences of grappling with these difficulties encompass a spectrum of sentiments that influence nursing students’ resilience, particularly when confronted with the demands of academic and clinical requirements.

One participant shared her experience of feeling unable to act when she recounted a challenging attempt to draw blood from a patient. She expressed feelings of guilt toward the patient and sadness regarding her perceived failure in the procedure:

*One day, I was assigned to draw blood on a patient, and it was very difficult to find her blood vessels. I intended to do it at once because I didn’t want the patient to get hurt several times, but it wasn’t successful. So, the nursing instructor stepped in and did it. I was feeling guilty with the patient and sad about my failure.*

(P4)

Another participant, reflecting on her inability to perform a nursing procedure successfully on the first attempt, expressed a sense of disappointment and self-criticism. The experience elicited feelings of discouragement and led her to question her own capabilities and competence in the field. The resulting emotional impact manifested as a period of introspective silence during which she acknowledged a decline in self-esteem and an overall sense of being discouraged by the perceived failure:

*….I feel really upset with myself that I could not do that nursing procedure at the first attempt during today’s clinical practice. I thought I would have done it better, and it’s kind of discouraging to comprehend that I failed. That experience really made me question myself and my competencies. (Being quiet for a while) ….It made me feel quite down and low self-esteem.*

(P14)

One participant described reaching a point of emotional exhaustion and distress during his clinical placement. Overwhelmed by the challenges, he sought comfort in confiding in his mother. During the conversation, he conveyed a sense of desperation, expressing a desire to discontinue his nursing studies:

*Once, I remember. I was exhausted and called my mother after leaving the ward that day. I told her that I couldn’t do it anymore and wanted to quit. I was crying while talking to her.*

(P5)

As the quote indicates, nursing students encounter a range of challenges that extend beyond procedural tasks and include administrative duties and emotional strains. Feelings of guilt, sadness, and self-doubt arise when students face difficulties in executing procedures, leading to a decline in resilience and self-esteem.

#### 3.1.3. Being Exposed to Diverse Encounters

The experiences of being exposed to diverse challenges during clinical practice impact nursing students’ resilience. One primary challenge nursing students face is the constraint of time, which is the nature of nursing education. Additionally, witnessing patients’ suffering can evoke a range of emotional responses, leading to feelings of uselessness, worry, and emotional exhaustion. Managing self-imposed and external expectations adds another layer of complexity, as nursing students may struggle with feelings of inadequacy and the fear of not meeting the standards set by their nursing instructors and peers.

One participant shared his experience regarding the unexpected emotional impact of clinical practice. Despite self-identifying as “strong”, he described an internal conflict triggered by witnessing patients’ suffering. The persistent nature of these emotions, “difficult to get rid of after each shift”, suggests a challenge to his previously perceived resilience. Furthermore, anticipatory anxiety (“worried in advance”) reveals the depth of emotional engagement and potential long-term effects on well-being:

*Normally, I am a strong person, and I did not expect that I would feel emotional by seeing patients in pain and suffering. However, when it came to clinical practice and witnessed so many patients suffering with their severe conditions, it was so difficult for me to get rid of those feelings after each shift. It was like my resilience got tested. Sometimes, I was even worried in advance that what may come today when I went to the ward tomorrow.*

(P15)

Being exposed to emotional challenges was addressed in the following quote given by one participant. She navigated personal empathy and professional demeanor when interacting with the concerned family members of her patient. She described feeling emotionally drained after comforting families with severe cases and struggled to compartmentalize their sadness and maintain professionalism. Her statement further emphasizes the ongoing emotional impact, with instances of rumination and sleep difficulties persisting beyond the workday:

*The first time I had to comfort family members of my patient that were extremely worried about his severe conditions, it drained my energy almost out of myself as I was feeling very sad for them. As a nursing student, I must talk professionally with them and tried to separate between my emotions from my duty. Sometimes, I was still thinking about this kind of situation after finish the shift, and had a hard time to sleep.*

(P18)

Another participant expressed feelings of inadequacy and self-doubt in the face of high expectations and perceived the pressure to perform flawlessly. The fear of revealing a lack of experience is highlighted by her statement regarding questioning whether others recognize her inexperience:

*Facing the high expectations during the first clinical practice made me feel like I don’t belong here. There’s a continual pressure regarding performing perfect nursing procedures, and making no mistake. Sometimes, I had a question pop out on my head Nobody knows that this is my first clinical practice? Sometimes, I felt that everyone expected myself to be able to do everything at the first day.*

(P21)

As the quote mentions, time constraints and high expectations inherent in nursing education pose substantial challenges. Witnessing patient suffering triggers complex emotional responses, including feelings of inadequacy and emotional exhaustion. Negotiating self-imposed and external expectations adds further complexity, fostering self-doubt and the fear of failure. The participants recounted emotional struggles, revealing the depth of their impact on resilience. Witnessing patient distress prompts internal conflicts while managing family interactions induces an emotional drain. Fear of inadequacy and the pressure to excel exacerbate emotional strain. These narratives underscore the multifaceted nature of nursing students’ challenges in clinical settings, shaping their resilience and emotional well-being.

### 3.2. Experiences of Meaningfulness

The overarching theme of “experiences of meaningfulness” regarding nursing students’ resilience in the context of clinical education discloses a multifaceted narrative delving into the intricate interaction between the challenges faced by nursing students and their resilience, which enables them to navigate these complexities. Within this theme, the sub-themes “restoring strength through social interactions” and “engaging in positive transformation” expound on the role of supportive structures in cultivating resilience among nursing students.

#### 3.2.1. Restoring Strength through Social Interactions

Nursing education is a multifaceted and integral aspect that influences nursing students’ resilience. Engaging in open communication with family, seeking encouragement from nursing instructors and patients, actively participating in the healthcare team, and fostering relationships with peers all contribute to restoring the strength that supports nursing students’ resilience during clinical practice. The impact of having a kind, friendly, and understanding nursing instructor is highlighted by the nursing students. Through shared experiences with peers, self-reflection, and participation in enjoyable activities, nursing students bolster their resilience and experience a sense of motivation, confidence, personal growth, and belonging.

One participant highlighted the crucial role of family support in maintaining resilience during clinical practice. She described regular communication with her mother and sister as a source of positive energy, enabling her to “keep fighting” through challenges. This suggests that family encouragement is an important factor in continuously building resilience:

*…family always help fill up positive energy for me. Whenever I call my mother and my sister or go home. My mother and my sister always tell me to keep fighting (laughs). Hmm…so, it’s like my resilience is filled up.*

(P1)

Another participant described clinical practice as physically and mentally demanding, leading to feelings of exhaustion and weakness. However, great support from family, peers, patients, and, especially, a kind and encouraging nursing instructor were significant sources of motivation and energy replenishment:

*It can be tiring during clinical practice. Many times, I felt like I had no energy left. I mean...it’s like my body is exhausted, and my mind is weak. I couldn’t go home during clinical practice, but I talked to my family over the phone or LINE. (Stop to think for a while) During the day, a nursing instructor is the closest person to all nursing students. If my nursing instructor talks to us with a kind voice, gives us support for what we do and does not blame us in front of patients, all these positive things help to fill up our energy and are our best support. I remember feeling so great when my nursing instructor said I’m so impressed with what you did! Well done! And she patted my shoulders while saying that. It’s like she recharged my battery. (Smile)*

(P28)

The experience of receiving support from patients was highlighted. One participant shared the unexpected impact of patient interactions on her emotional well-being during clinical practice. While initially focused on the emotional support she provided to patients, she revealed how their reciprocated concern was a source of encouragement for her own emotional resilience. This dynamic was described as a “topping up” of encouragement:

*Yes, the patients. As I mentioned earlier about the patient that I took care on the hospital ward. Whenever I took care of him, he always said thank you. He also asked me about “How are you learning? Have you studied too hard? How about the work? Are you scared?”. It’s like I got the encouragement from the patients. Although it was like I could vented my feelings when I talked to the patients, but I surely did not tell them everything or all problems. I think, sometimes, talking to patients can make us feel like topping up the encouragement.*

(P2)

Referring to the quotes above, social interactions emerge as a vital source of strength and resilience among the participants. Fostering connections with family, peers, nursing instructors, patients, and healthcare professionals enables participants to create a network of encouragement and shared experiences. The quotes highlight that social interactions provide a safe space to process challenges and offer practical advice. This sense of community becomes a wellspring of emotional support and promotes a sense of belonging and purpose, ultimately allowing the participants to manage the complexities of clinical practice with renewed strength and resilience.

#### 3.2.2. Engaging in Positive Transformation

In the clinical practice environment, nursing students embark on a transformative journey encapsulated by engaging in positive transformation. This sub-theme encompasses a broad range of important elements, including accomplishment, gratitude, teamwork within the healthcare domain, empowerment, energy acquisition, purpose-driven activities, success recognition, and self-care.

From the viewpoint of one participant, a sense of fulfillment could be derived from actively contributing to a patient’s recovery. She underscored the gratifying experience of being acknowledged by patients who, upon expressing gratitude to all staff at the clinical placement, bestowed a heightened sense of purpose on her educational pursuit in nursing:

*It was such a good feeling to be one of the people that helped a patient getting through their challenging time in life. And, when they share their gratitude to everyone on the ward before going home with their relatives, it always builds up a sense of purpose to the journey of my nursing education.*

(P10)

Another participant tried to identify and acknowledge her achievements. Regardless of the scale, she maintained a practice of self-affirmation, vocalizing commendations such as “You did it! Great job!”. This deliberate recognition of accomplishments was supplemented by special ceremonies, exemplified by indulging in flavored beverages, such as iced green tea, and sharing experiences with family through communication. Additionally, she perceived these affirmations and celebratory acts as instrumental in fortifying personal resilience and cultivating a proactive readiness to confront forthcoming challenges in the clinical setting:

*During the chaotic time of clinical practice, we may not recognize our small achievements. For me, I had tried to recognize all success. It doesn’t matter that it’s small or big. I always acknowledge it by saying to myself that You did it! Great job! And I try to find time to celebrate by doing simple things, such as buying myself favorite drinks—Iced Green Tea, calling my family to tell them about what I did, etc. This help support my resilience and prepare myself to prepare for the upcoming challenges.*

(P27)

One participant, reflecting on her first clinical practice, recounted a transformative experience characterized by enriching engagement in patient care. Despite initial apprehension, she found thoughtful fulfillment in successfully administering treatment and evoking smiles of gratitude from both the patients and their relatives. The sense of accomplishment derived from these interactions was akin to the administration of an astonishing “vaccine” of positive energy:

*Although, I might feel a bit scared before starting my first clinical practice, there’s something amazingly enriching about providing care for actual patients. When I was successful with providing treatment for my patients, the patient and his relatives looked at me with a big smile. I was having the feelings of accomplishment. It was like I was given a shot of miracle vaccine called positive energy.*

(P6)

Reflecting on the previous quote, engaging in clinical practice evokes a spectrum of emotions in nursing students, from initial apprehension to reflective fulfillment. The firsthand experience of providing care to patients brings a sense of accomplishment and invigorates them with positive energy, akin to a “miracle vaccine”. Moreover, despite the chaos of clinical settings, the participants recognized and celebrated even the smallest achievements, reinforcing their resilience. Simple acts of self-recognition and celebration, such as indulging in favorite drinks or sharing successes with family, fortified their resolve. Contributing to patients’ well-being imbued their nursing education journey with a sense of purpose, reinforcing their commitment to the profession.

## 4. Discussion

In the demanding nature of nursing education, nursing students often navigate complex challenges that shape their experiences, influence their professional identities, and impact their overall well-being. This study explores the experiences of nursing students’ resilience regarding challenges and supports for their resilience during clinical practice, focusing on two overarching themes: “experiences of vulnerability”, which encapsulates the multifaceted dimensions of vulnerability faced by nursing students, and “experiences of meaningfulness”, which highlight the essential role of support systems and progress in fostering nursing students’ resilience.

Embarking on the intricate journey of nursing education, nursing students frequently encounter challenges intertwined with vulnerability [39]. Within this context, the sub-theme “navigating uncertainty” delves into the complex experiences of doubt and discomfort among nursing students when confronted with unfamiliar situations or knowledge gaps, highlighting the intricate cognitive and emotional dimensions of their learning journeys. As novices in the healthcare team, nursing students deal with the delicate equilibrium between acquiring essential knowledge and acknowledging the disconcerting awareness of their limitations [40], thereby shaping the trajectory of their resilience. This vulnerable experience, entangled in the academic and experiential context of nursing education, necessitates exploring its implications for nursing students’ professional development [41].

The complex and dynamic nature of clinical practice presents nursing students with numerous challenges, impacting their resilience and potentially hindering their professional development. Among these challenges, the inherent uncertainty of clinical situations and the need for self-awareness influence nursing students’ resilience. Uncertainty in clinical practice stems from various factors, including unforeseen patient complications, ambiguous diagnoses, and uncertainty related to the educational process [42,43]. This uncertainty can trigger feelings of anxiety, self-doubt, and inadequacy, potentially hindering nursing students’ ability to make sound decisions and effectively manage challenging situations [44]. Balancing this uncertainty with self-awareness is critical for building resilience. Self-awareness, which includes self-reflection, emotional intelligence, and an understanding of one’s limitations and strengths, enables nursing students to identify their emotional reactions to uncertainty and to foster effective coping strategies. [45]. A unitary caring science resilience-building model highlighted a positive correlation between nursing students’ resilience and their self-awareness in clinical education [10]. Dealing with uncertainty and balancing self-awareness are intricately linked challenges that impact nursing students’ resilience during clinical practice [46]. Therefore, self-awareness interventions should be integrated into nursing education to enhance resilience. Consequently, nursing instructors can empower nursing students to navigate uncertainty effectively, fostering resilience and paving the way for confident and competent nursing professionals [47,48].

The sub-theme “transcending professional struggles” illuminates the complex challenges stemming from nursing students’ perceived inability to effectively transfer theoretical knowledge into practical scenarios. This challenge of being unable to perform engenders a sense of vulnerability among nursing students within their clinical practice [49]. As these students strive to bridge the gap between classroom learning and clinical training, they grapple with the profound implications of their actions on patient outcomes, which further contributes to the complexity of their vulnerability [50,51]. This aspect underscores the critical juncture at which theoretical understanding meets the demands of clinical reality, warranting a thorough examination to elucidate the dynamics shaping nursing students’ perceptions of their competence and resilience in clinical settings [40].

The application of theoretical knowledge in practical settings presents challenges that contribute to a deeper understanding of resilience in nursing education. Recognizing these obstacles allows nursing instructors to develop precise methodologies designed to support nursing students in effectively translating theoretical concepts into practical skills, thus bridging the gap between these two areas [49]. This could involve incorporating more simulation-based learning, case studies, and reflective practice exercises into the curriculum. These approaches allow students to practice applying their theoretical knowledge in controlled environments, building their confidence and resilience before entering real clinical settings [35]. In addition, fostering strong mentorship relationships between nursing instructors, clinical preceptors, and nursing students provides a supportive framework for navigating the complexities of translating theory into practice, ultimately enhancing nursing students’ resilience [48]. In the context of nursing education, the enhancement of resilience among nursing students is intricately linked to their ability to transcend professional struggles through the creative use of the self [52]. Recent research underscores the significance of creativity in fostering resilience, as it enables nursing students to tackle challenges and setbacks in clinical practice with adaptability and resourcefulness [15]. As highlighted in a unitary caring science resilience-building model, by harnessing creativity, nursing students can transcend conventional problem-solving methods and skills, tapping into their essential resourcefulness to address complex clinical dilemmas and interpersonal conflicts [10]. Furthermore, a recent study highlighted the positive correlation between creative self-expression and psychological well-being [53], suggesting that fostering creativity among nursing students can not only enhance their resilience but also promote overall mental health.

One significant challenge nursing students face in clinical practice is navigating the complexity of adversities, as indicated in the sub-theme “being exposed to diverse encounters”. Encounters with patients from different backgrounds, cultures, and health realities can push nursing students outside their comfort zones, potentially triggering feelings of vulnerability. However, when approaching these encounters with mindful self-awareness, they can become powerful catalysts for fostering resilience and professional growth [10]. Exposure to diverse encounters opens a doorway to evoking nursing students’ consciousness. This heightened awareness of different perspectives, needs, and experiences challenges students to examine their biases and limitations.

This study underscored the importance of diverse clinical placements in fostering resilience among nursing students. Exposure to a wide range of patient populations and healthcare settings allows students to develop adaptability, cultural competence, and empathy, which are key components of resilience [21]. Nursing education programs can intentionally structure clinical rotations to ensure students have opportunities to engage with diverse patient populations. Moreover, incorporating reflective practice and debriefing sessions can help nursing students process and learn from these diverse encounters, promoting self-awareness and personal growth [45]. Embracing the challenges of diverse clinical experiences as opportunities for learning and growth, nursing education fosters the cultivation of more resilient practitioners prepared to navigate the complexities of contemporary healthcare. Diverse clinical placements enhance clinical skills and cultivate cultural competency and empathy, which are key resilience builders [54]. Recognizing the limitations of their current knowledge and understanding motivates nursing students to actively seek resources and learning opportunities that enhance their learning ability [55]. Ultimately, exposure to diverse encounters, rather than being seen as a barrier, can act as a springboard for self-awareness and self-learning. Cultivating these aspects empowers nursing instructors to prepare students to navigate the complexities of clinical practice with confidence, empathy, and a resilient essence, ensuring their readiness to thrive in an increasingly diverse healthcare system.

Contrasting the challenges of vulnerability, the theme of “experiences of meaningfulness” illuminates crucial sources of support and progress that contribute to the resilience of nursing students. This theme underscores the intricate interplay between the adversities nursing students encounter during clinical practice and the resilience they develop to navigate these challenges. Within this overarching theme, two sub-themes, “restoring strength through social interactions” and “engaging in positive transformation”, elucidate the supportive structures and adaptive strategies that underpin resilience in nursing students.

In the context of supporting nursing students’ resilience during clinical practice, the first sub-theme, “restoring strength through social interactions”, emphasizes the importance of supportive resources related to family members, peers, nursing instructors, nursing preceptors, and patients. The multifaceted support system provided by these interactions plays a crucial role in strengthening resilience in demanding clinical environments. Engaging in meaningful social interactions allows nursing students to derive emotional sustenance, garner coping strategies, and promote a sense of belonging, all of which are vital components of resilience [8,56]. The positive influence of family support, for instance, serves as a continuous source of motivation and energy, enabling students to persevere through the rigors of clinical practice. Similarly, instructors’ support, encouragement, and understanding positively impact resilience among nursing students during clinical practice [57]. Interactions with patients, who often express gratitude and concern to nursing students, also provide an unexpected but powerful source of emotional support, highlighting the reciprocal nature of caregiving relationships [58].

Moreover, nurturing interpersonal and intersubjective connections reinforces the depth of relational experiences that contribute to resilience-building. This dynamic is well illustrated in the unitary caring science resilience-building model, which underscores the importance of interconnectedness and mutual support [10]. Recent research corroborates these findings, demonstrating how fostering strong interpersonal bonds not only provides emotional support but also facilitates collaborative learning, constructive feedback exchange, and shared problem-solving [59]. These interactions collectively fortify nursing students’ capacity to navigate the complexities of clinical practice with resilience and efficacy. Integrating these concepts within educational frameworks can thus serve as a cornerstone in promoting future nursing professionals’ well-being and professional development. Embedding opportunities for meaningful social interactions within the curriculum enhances students’ resilience in nursing education programs, ultimately contributing to a more robust and compassionate healthcare workforce.

The second sub-theme, “engaging in positive transformation”, highlights the adaptive response to adversity, wherein nursing students cultivate resilience by actively seeking opportunities for personal growth and professional development. This transformative process is intricately linked with embracing loving-kindness for oneself and others, fostering a compassionate and supportive internal environment [10]. Additionally, valuing forgiveness and releasing negativity contribute to resilience by freeing individuals from resentment and promoting psychological flexibility [60]. This aspect of resilience allows nursing students to maintain a positive outlook and adaptability, essential traits for thriving in challenging clinical settings. Furthermore, maintaining faith and hope is a resilient mindset that instills optimism, perseverance, and a sense of purpose amidst challenges. These attributes are crucial for nursing students as they navigate the uncertainties and pressures of clinical practice. Integrating these concepts into nursing education empowers nursing instructors to prepare nursing students to approach their professional responsibilities with compassion and willpower. This holistic approach to resilience not only prepares nursing students to handle instant challenges but also fosters long-term professional growth and satisfaction. Ultimately, nurturing resilience through positive transformation ensures the development of a capable and compassionate nursing workforce ready to deliver high-quality patient care [8,16].

The theme of “experiences of meaningfulness” provides valuable insights into the factors that support and enhance the resilience of nursing students during clinical practice. This theme underscores the multifaceted nature of resilience and its critical role in nursing education by emphasizing the importance of social interactions and positive transformations. Integrating these elements into educational frameworks is essential for cultivating a resilient and proficient nursing workforce capable of meeting the demands of the healthcare environment with empathy and professionalism.

### Strengths and Limitations of the Study

A notable strength of this research is its qualitative design, which permits an in-depth examination of nursing students’ experiences and the factors influencing their resilience in clinical settings. The application of semi-structured interviews facilitated the gathering of rich, personal insights that quantitative approaches might not have captured. Additionally, this study offers a thorough thematic analysis, revealing the complex and multifaceted nature of nursing students’ experiences. This study achieved data saturation with 28 interviews, indicating that the sample size was sufficient to capture a comprehensive range of experiences and perspectives [37]. In addition, this study followed a rigorous data analysis process using Braun and Clarke’s thematic analysis approach, ensuring a systematic and transparent analysis of the data. Identifying both the challenges faced and the sources of support, the research offers a balanced perspective that can inform future educational strategies and interventions. Moreover, the study addressed an important topic in nursing education, highlighting the significance of resilience and the need for comprehensive support systems to foster resilience among nursing students during clinical practice. Investigating how nursing students confront and overcome adversities enhances our understanding of the factors that promote the development of resilient and competent nursing professionals.

Despite the valuable insights provided, this study has several limitations. This study was conducted in a specific context (nursing colleges in Thailand under the Faculty of Nursing at the Praboromarajchanok Institute), which may limit the generalizability of the findings to other nursing education settings or cultural contexts. Additionally, the qualitative approach, while rich in detail, restricts the generalizability of the findings to a wider population. Moreover, this study’s reliance on self-reported data introduces potential recall bias or social desirability bias. Participants might have given responses they deemed favorable or might not have accurately remembered their experiences. Furthermore, the shift to digital platforms for interviews due to COVID-19 restrictions, though necessary, may have influenced the depth and nature of the data collected. Face-to-face interactions typically yield more nuanced and richer data, and the digital format may have constrained certain aspects of the participants’ expressions and interactions. In addition, the study sample was predominantly female, which may not fully capture the experiences and perspectives of male nursing students.

## 5. Conclusions

In conclusion, the themes of “experiences of vulnerability” and “experiences of meaningfulness” provide novel insights into the challenges nursing students face during clinical practice and the support mechanisms essential for fostering resilience. Navigating uncertainty, transcending professional struggles, and exposing oneself to diverse encounters encapsulate the multifaceted nature of the vulnerability experienced by nursing students. These experiences underscore the importance of comprehensive support systems that address both personal and professional vulnerabilities. Integrating principles from caring theory into these support systems can further amplify resilience by emphasizing the significance of compassionate patient care and nurturing a deep sense of connection and empathy among nursing students. At the same time, restoring strength through social interactions and engaging in positive transformation highlight the significance of fostering a sense of belonging within the nursing community. Nursing instructors and other stakeholders can significantly enhance nursing students’ resilience and well-being by cultivating supportive environments that promote collaboration, empathy, and mentorship, all grounded in the principles of caring theory. This holistic approach, rooted in understanding vulnerability, meaningfulness, and caring, is vital for preparing nursing students to face the complexities of clinical practice and emerge as competent and resilient healthcare professionals.

## Figures and Tables

**Table 1:** Interview guide.

Interview Guide
Introductory interview: It encompassed a series of broad inquiries designed to cultivate a comfortable atmosphere for the participants and establish rapport. When did you complete your last clinical practice? Where did you go for your last clinical practice?
Main interview: It focused on the participants’ experiences of challenges and support for their resilience during clinical practice. Please tell me about a situation in your clinical practice when you encountered adversities. What do you think…/How do you feel about…the situation? How did you act in the situation? How do you handle difficulties or negative experiences in clinical practice? Who or what helped you overcome adversity during clinical practice? In your opinion, what do nursing students need in order to overcome obstacles and feel better during clinical practice?
Ending interview: wrapped up the interview. Is there anything else that you would like to add?

**Table 2:** Main themes and sub-themes.

Main Themes	Sub-Themes
Experiences of vulnerability	Navigating uncertainty Transcending professional struggles Being exposed to diverse encounters
Experiences of meaningfulness	Restoring strength through social interactions Engaging in positive transformation

## Data Availability

The datasets used and analyzed during the current study are available from the corresponding author upon reasonable request.

## Public Involvement Statement

No public involvement in any aspect of this research.

## Guidelines and Standards Statement

This manuscript was drafted against the Consolidated criteria for reporting qualitative research (COREQ): for qualitative research. (https://www.equator-network.org/reporting-guidelines/coreq/, accessed on 7 February 2024).
